# Psychopharmacological Medication Has No Influence on Vitamin Status After Bariatric Surgery in Long-term Follow-up

**DOI:** 10.1007/s11695-020-04698-8

**Published:** 2020-05-22

**Authors:** Hannes Beiglböck, Alexander Kautzky, Paul Fellinger, Tamara Ranzenberger-Haider, Bianca Itariu, Thomas Wrba, Gerhard Prager, Alexandra Kautzky-Willer, Peter Wolf, Michael Krebs

**Affiliations:** 1 Department of Internal Medicine III, Division of Endocrinology and Metabolism, Medical University of Vienna, Währinger Gürtel 18-20, 1090 Vienna, Austria; 2 Department of Psychiatry and Psychotherapy, Division of Social Psychiatry, Medical University of Vienna, Währinger Gürtel 18-20, 1090 Vienna, Austria; 3 IT Systems and Communications, Medical University of Vienna, Währinger Gürtel 18-20, 1090 Vienna, Austria; 4 Division of General Surgery, Department of Bariatric Surgery, Medical University of Vienna, Währinger Gürtel 18-20, 1090 Vienna, Austria

**Keywords:** Bariatric surgery, Vitamins, Psychiatric medication

## Abstract

**Context:**

A substantial number of patients undergoing bariatric surgery are prescribed psychopharmacological medication. However, the impact of concomitant psychopharmacological medication on the frequency of relevant vitamin deficiencies in postoperative follow-up is not known.

**Methods:**

Five hundred twenty-four patients with obesity who underwent bariatric surgery (January 2004 to September 2018) with follow-up of at least 12 months, were included in retrospective analysis. Postoperative follow-up visits between January 2015 and September 2019 were analyzed. Anthropometric and laboratory data were analyzed at the first documented follow-up visit after on average 39.5 ± 37.3 months and at every following visit during the observation period. Patients with prescribed psychopharmacological drugs (PD) were compared with patients without (control group, CON).

**Results:**

Psychopharmacological medication was documented in 25% (132) of patients. In 59 patients documented prescription of more than one psychiatric drug was found, whereas psychopharmacological monotherapy was found in 73 patients. Frequencies of vitamin deficiencies were comparable between PD and CON (vitamin A: *p* = 0.852; vitamin D: *p* = 0.622; vitamin E: *p* = 0.901; folic acid: *p* = 0.941). Prevalence of vitamin B_12_ deficiency was rare (6% CON, 1% PD) but was significantly higher in CON (*p* = 0.023). A comparison of CON and POLY also showed no significant differences between the groups concerning prevalence of vitamin deficiencies.

**Conclusions:**

Intake of psychopharmacological medication is highly prevalent in patients after bariatric surgery. Patients with psychopharmacological medication, who participate in structured follow-up care after bariatric surgery, are not at higher risk for vitamin deficiencies compared with controls.

## Background

Bariatric surgery is the most effective therapy for patients with morbid obesity, leading to substantial weight loss. This weight-loss improves metabolic health and decreases mortality. Thus, metabolic surgery is an established therapy to combat comorbidities linked with severe obesity [1–3].

Morbid obesity has a fundamental impact on mental health and is associated with high prevalence of depression disorders [4]. Regarding long-term outcomes in patients with psychiatric diseases diagnosed before undergoing bariatric surgery, the evidence is conflicting. Bariatric surgery might enhance specific risk behavior leading to increased incidence of suicide as well as higher risk of non-fatal self-harm and alcohol abuse [5–7]. While symptoms of depression have been reported to improve in the short-term to mid-term following bariatric surgery [8], another study indicates that bariatric surgery is associated with an increased risk of developing depression [9]. In synopsis, there is evidence for an increase in symptom severity as well as diagnosis of psychiatric disorders in the long-term after bariatric surgery [10–14], including an increased risk of completed suicide as well as non-fatal self-harm [7, 15].

Following bariatric surgery, patients’ compliance and the adherence to regular intake of vitamin and dietary mineral supplementation is essential to avoid potential adverse sequelae of malabsorption and malnutrition. A high number of patients, after bariatric surgery, have problems with the intake of the recommended supplements due to different reasons (e.g., high number of supplements, difficulties in remembering the prescriptions, and high costs). [16–18]. In patients with psychiatric disorders, non-adherence to recommended psychopharmacological medication can be expected in up to 50% of long-term prescriptions and thus remains a significant challenge [19]. Adherence to recommended supplementation after bariatric surgery might be reduced due to symptoms of psychiatric disorders (e.g., reduced motivation, reduced self-care, cognitive impairment, eating disorders, or substance use disorders).

We therefore hypothesized that the presence of a psychiatric disorder indicated by psychopharmacologic therapy among patients after bariatric surgery might be associated with poorer adherence to recommended vitamin supplementation and therefore leading to more frequent vitamin deficiencies compared with controls. Furthermore, mental disorders indicated by the usage of antidepressants and antipsychotics during postoperative follow-up might influence the postoperative benefits of bariatric surgery and/or patient safety. Therefore, the aim of this retrospective analysis was to evaluate the impact of psychopharmacological medication intake on the postoperative follow-up with respect to vitamin levels.

## Methods

**Fig. 1:**
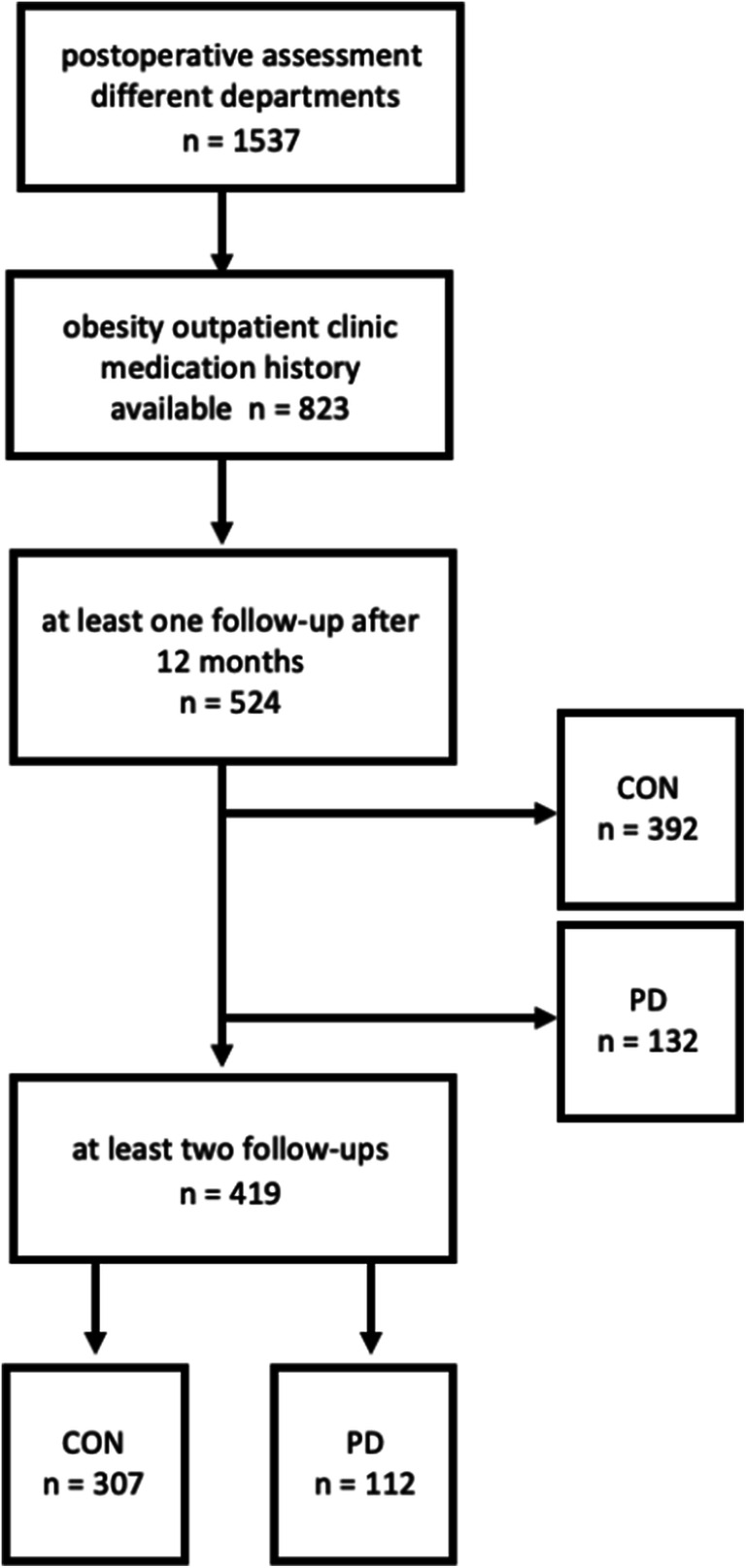
Flowchart of the enrollment process; CON, control group; PD, intake of psychopharmacological drugs (documented at least at one visit)

Records of all patients who gave their informed consent to data collection in routine care at the obesity outpatients’ clinics of the Medical University of Vienna, Department of Medicine III, Division of Endocrinology and Metabolism, from January 2015 to September 2019 were analyzed. The study protocol was approved by the ethics committee of the Medical University of Vienna. Structured postoperative care included a standardized comprehensive set of laboratory analyses and medication history. The main hypothesis, definition of groups, and main outcome variables were defined before data analysis. The inclusion criteria for men and women were history of bariatric surgery for morbid obesity with a follow-up period of at least 12 months and full availability of anthropometric data (height, weight, and body mass index (BMI)) as well as laboratory parameters, including vitamin A, 25-hydroxy vitamin D, vitamin B12, vitamin E, and folic acid. Five hundred twenty-four patients (76% women, 24% men) met these criteria and were included in the analysis (Fig. 1). Anthropometric data as well as levels of vitamins, parameters of glucose and lipid metabolism (HbA1c, triglycerides (TG), total cholesterol, high-density cholesterol (HDL), liver enzymes (GGT, ASAT, ALAT), and concentrations of albumin and parathyroid hormone (PTH) were analyzed postoperatively at every follow-up visit at the obesity outpatients’ clinic. Conditions of vitamin deficiencies were defined according to assay specific reference values (see Table 1 and www.kimcl.at): hypovitaminosis D was defined as 25-hydroxy vitamin D levels below 75 nmol/l; hypovitaminosis B_12_ was as vitamin B_12_ levels below 145 pmol/l; hypovitaminosis A was defined as vitamin A levels below 1.05 μmol/l; hypovitaminosis E was defined as vitamin E levels below 12 μmol/l; deficiency in folic acid was defined as levels of folic acid below 9.53 nmol/l. Patients were grouped according to the prescription of psychopharmacological medication (antidepressants, antipsychotics, tranquilizers, benzodiazepines, Z-drugs, stimulants, mood stabilizers) documented over all available follow-up visits at the outpatient’s clinic in PD vs CON. Additionally, documented recommended vitamin and mineral supplementation was analyzed and coded into different groups: standard multivitamins (e.g., Supradyn®, Centrum®…), special multivitamins for post-bariatric surgery purpose (e.g., Bariatric Advantage®, Fit for Me®…), extra vitamin A supplement, extra vitamin D supplement, extra calcium supplement, extra iron supplement, and extra vitamin B_12_ supplement. All laboratory parameters were assessed by using routine laboratory methods (www.kimcl.at). Exploratory statistical analysis was performed using SPSS (IBM, version 26) and Microsoft Excel (Microsoft, 2019). Normal distribution was checked by data visualization. Student’s *t* tests and chi-square tests were used to compare data between the different groups. For not normally distributed data, Mann-Whitney *U* tests were performed. Data was analyzed using exploratory statistical analysis. Data is given as means ± standard deviation. Statistical significance level was set at *p* < 0.05.

**Table 1:** Anthropometric data and laboratory parameters in all included patients at first visit and last postoperative follow-up visit during the observation period January 2015 to September 2019

	First visit		Last visit	
	CON	PD	CON	PD
*n*	392	132	307	112
Sex (female/male)	292/100	106/26	226/81	91/21
Follow-up visits	-	-	4.3 ± 2.7	4.6 ± 3.1
Follow-up since surgery (months)	39.7 ± 38.6	38.8 ± 33.6	56.1 ± 36.4	60.9 ± 38.0
Age (years)	45.2 ± 12.6	49.8 ± 11.0^#^	47.2 ± 12.7	51.9 ± 11.0^#^
Body weight (kg)	84.1 ± 18.3	84.0 ± 20.3	84.7 ± 17.3	83.6 ± 20.8
BMI (kg/m^2^)	29.6 ± 5.6	29.8 ± 6.2	29.9 ± 5.2	30.0 ± 6.3
BMI before OP	45.9 ± 7.1	45.4 ± 7.7	45.6 ± 6.8	44.6 ± 7.4
Change in BMI	− 16.3 ± 6.1	− 15.6 ± 7.2	− 15.7 ± 6.0	− 14.6 ± 6.9
Gastric bypass (*n*; %)	147; 38%	58; 44%	123; 40%	52; 46%
One-anastomosis gastric bypass (*n*; %)	192; 49%	54; 41%	152; 50%	48; 43%
Sleeve gastrectomy (*n*; %)	39; 10%	14; 11%	24; 8%	11; 10%
Other procedures (*n*; %)	14; 3%	6; 4%	8; 2%	1; 1%
Antidepressants (*n*; %)	-	102; 77%	-	86; 77%
Antipsychotics (*n*; %)	-	26; 20%	-	21; 19%
Other psychotropic drugs (*n*; %)	-	80; 60%	-	70; 63%
Prescribed Supplements	100%	100%	100%	100%
Standard multivitamin supplement	50%	50%	52%	53%
Special “bariatric” multivitamin supplement	48%	46%	47%	41%
Extra vitamin D supplement	82%	84%	83%	86%
Extra calcium supplement	61%	64%	64%	62%
Extra vitamin A supplement	7%	11%	6%	10%
Extra iron supplement	30%	33%	31%	35%
Extra vitamin B_12_ supplement	34%	35%	37%	37%
ASAT (< 50 U/l)	26.5 ± 13.5	26.5 ± 10.4	25.7 ± 10.7	25.6 ± 11.1
ALAT (< 50 U/l)	30.4 ± 19.5	30.2 ± 16.7	28.8 ± 15.3	28.9 ± 14.7
GGT (< 60 U/l)	22.1 ± 30.1	26.7 ± 34.1	19.3 ± 21.9	23.5 ± 21.7
Albumin (35–52 g/l)	42.8 ± 3.2	42.1 ± 3.1^#^	43.2 ± 3.3	43.1 ± 3.2
Creatinine (0.5–1.2 mg/dl)	0.7 ± 0.2	0.8 ± 0.4^#^	0.7 ± 0.3	0.8 ± 0.4^#^
TG (< 150 mg/dl)	85.5 ± 37.2	98.9 ± 49.2^#^	88.0 ± 40.8	99.2 ± 54.3^#^
Total cholesterol (< 200 mg/dl)	161.1 ± 30.8	166.0 ± 37.1	164.8 ± 29.6	167.1 ± 32.9
HDL-cholesterol (> 55 mg/dl)	60.3 ± 13.8	59.0 ± 16.7	62.9 ± 15.3	60.2 ± 15.2
HbA1c (4–6%)	5.3 ± 0.7	5.5 ± 0.9^#^	5.3 ± 0.6	5.6 ± 1.0^#^
PTH (15–65 pg/ml)	54.5 ± 29.5	64.2 ± 59.8	57.7 ± 31.9	63.7 ± 41.4
25-OH-VitD3 (75–250 nmol/l)	61.4 ± 23.5	66.7 ± 28.7^#^	66.4 ± 26.7	68.0 ± 26.2
Vitamin A (1.05–2.45 μmol/l)	1.37 ± 0.36	1.47 ± 0.54^#^	1.44 ± 0.40	1.53 ± 0.55
Vitamin E (12–42 μmol/l)	24.0 ± 5.2	24.7 ± 6.7	24.3 ± 5.8	24.5 ± 6.5
Vitamin B_12_ (145–569 pmol/l)	411.3 ± 246.0	457.9 ± 249.8	474.7 ± 265.8	455.4 ± 241.8
Methylmalonic acid (73–271 nmol/l)	170.7 ± 130.6	211.4 ± 217.1	177.7 ± 146.3	213.2 ± 188.8
Folic acid (9.53–44.9 nmol/l)	24.5 ± 12.0	23.9 ± 12.1	25.3 ± 13.1	25.3 ± 13.3
Calcium (2.15–2.50 mmol/l)	2.27 ± 0.11	2.26 ± 0.11	2.28 ± 0.11	2.27 ± 0.10
Ferritin (15–400 μg/l)	71.5 ± 73.6	78.5 ± 72.6	62.0 ± 57.3	75.3 ± 71.7

*CON*, control group; *PD*, intake of psychopharmacological drugs (documented at least at one visit)

^#^*p* < 0.05 compared with CON

## Results

Of 1537 patients recorded in the database, 524 patients (76% women, 24% men) met all criteria including a minimum follow-up period of 12 months postoperatively.

Postoperative follow-up visits that occurred during the observation period between January 2015 and September 2019 were analyzed. The mean follow-up time at the first documented visit was 39.5 ± 37.3 months. Four hundred nineteen patients (80%) had at least one additional follow-up visit during the observation period. Mean frequency of follow-up visits of this cohort was 4.35 ± 2.8 visits. Frequencies of different surgical procedures were comparable between the groups (Table 1).

Psychopharmacological medication in documented medication histories was evident in 132 of 524 patients (25%; 80% women, 20% men) at a minimum of one follow-up during the observation period. Antidepressants were the most common drugs used (102 patients, 20%; 80% women, 20% men). Antipsychotic-therapy was present in 26 patients (5%; 77% women, 23% men) and 80 patients were taking other psychopharmacological drugs (15%; 80% women, 20% men). Fifty-nine patients (11%) were taking more than one psychopharmacological drug (81% women, 19% men).

At the first visit during the observation period, all 524 patients were found with documented recommendation for intake of mineral/vitamin supplements. Types of supplements as well as frequency of supplementation were not significantly different between CON and PD. Comparing last available follow-up visits for each patient, prescription of supplements was still comparable between CON and PD (Table 1).

At the first follow-up visit during the observation period, no differences between CON and PD regarding BMI, body weight, and change in body mass index were evident. However, age, triglycerides, HbA1c, and creatinine were significantly higher in PD. With respect to vitamin status, vitamin A and vitamin D were significantly higher in PD compared with CON (vitamin A (μmol/l): CON, 1.37 ± 0.36; PD, 1.47 ± 0.54, *p* = 0.039; vitamin D (nmol/l): CON, 61.4 ± 23.5; PD, 66.7 ± 28.7, *p* = 0.034).

The frequency of follow-up visits during the observation period and the body-mass index and the change in body mass index were well comparable with no differences between PD and CON at the last follow-up during the observation period. Nevertheless, patients’ age, creatinine, triglycerides, and HbA1c were still slightly higher in PD at last follow-up during observation period. However, serum concentrations of all vitamins did not show significant differences at the last follow-up. Data are given in detail in Table 1.

**Fig. 2:**
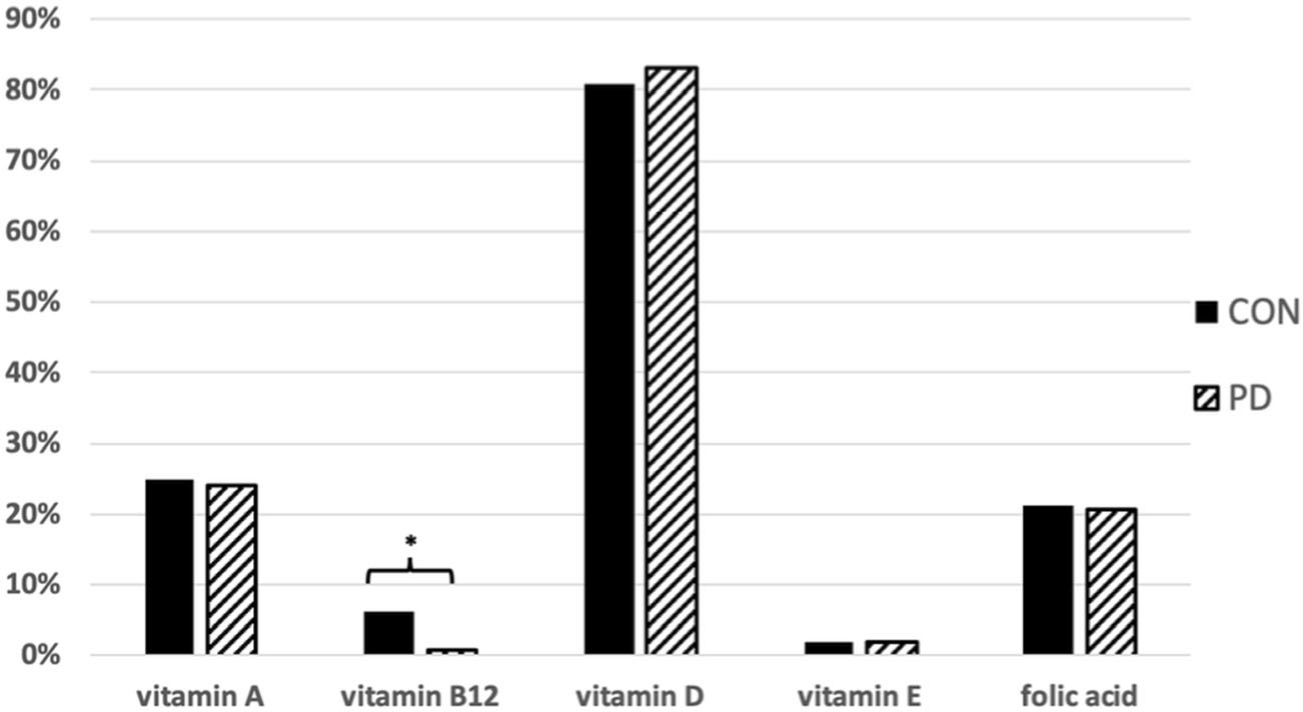
Prevalence of vitamin deficiency at a minimum of one follow-up visit; CON, control group; PD, intake of psychopharmacological drugs (documented at least at one visit); *p < 0.05

Vitamin A deficiency was documented throughout all visits during the observation period in 8% in CON and 9% in PD (Fig. 2). The frequency of at least one visit with vitamin A deficiency was not significantly different between the groups (CON, 25%; PD, 24%, *p* = 0.852). In 94% CON and 99% PD, levels of vitamin B_12_ were sufficient at all individual measurements during the observation period (Fig. 2). Prevalence of vitamin B_12_ deficiency, at a minimum of one follow-up during observation period, was significantly different between the groups (*p* = 0.023), being more prevalent in CON (6%) compared with PD (1%). Vitamin D deficiency was quite common with 53% in both groups at each individual visit during observation period (Fig. 2). Frequency of at least one visit with vitamin D deficiency was not significantly different between the groups (CON, 81%; PD, 83%, *p* = 0.622). Levels of vitamin E were sufficient at each individual visit during follow-up period in 98% in both groups (Fig. 2). Prevalence of vitamin E deficiency documented at least at one follow-up during observation period, was not significantly different between the groups (CON, 2%; PD, 2%, *p* = 0.901). Folic acid levels were insufficient at each individual measurement during observation period in 6% CON and 4% PD, respectively (Fig. 2). Frequency of at least one visit with folic acid deficiency was not significantly different between the groups (CON, 21%; PD, 21%, *p* = 0.941). Detailed data are given in Table 2.

**Table 2:** Percentage of follow-up visits with presence of vitamin deficiency

Percentage of visits below reference values	Vitamin A (1.05–2.45 μmol/l)		Vitamin B_12_ (145–569 pmol/l)		Vitamin D (75–250 nmol/l)		Vitamin E (12–42 μmol/l)		Folic acid (9.53–44.9 nmol/l)	
	CON	PD	CON	PD	CON	PD	CON	PD	CON	PD
0%	75.1%	75.8%	93.8%	99.1%	19.1%	17.0%	98.0%	98.2%	78.9%	79.3%
25%	4.9%	3.6%	1.7%	0%	3.6%	6.2%	0.4%	0.9%	3.3%	2.7%
50%	8.5%	8.1%	3.6%	0.9%	15.6%	16.0%	0.0%	0.9%	8.7%	9.0%
75%	3.9%	3.6%	0.6%	0%	8.8%	8.1%	0.3%	0%	3.2%	5.4%
100%	7.6%	8.9%	0.3%	0%	52.9%	52.7%	1.3%	0%	5.9%	3.6%

*CON*, control group; *PD*, intake of psychopharmacological drugs (documented at least at one visit

In a subgroup analysis, in which only patients with at least two follow-ups were included, patients taking more than one psychopharmacological drug (POLY) were compared with patients with only single psychopharmacological medication (SINGLE). Fifty-two patients taking at least 2 psychopharmacological drugs were classified as POLY, and 60 patients taking single psychopharmacological medication were classified as SINGLE. There were no significant differences regarding the frequency of at least one follow-up with vitamin deficiency between these groups (vitamin A: SINGLE 28%, POLY 19%, *p* = 0.261; vitamin B_12_: SINGLE 2%, POLY 0%, *p* = 0.350; vitamin D: SINGLE 83%, POLY 83%, *p* = 0.928; vitamin E: SINGLE 3%, POLY 0%, *p* = 0.184; folic acid: SINGLE 15%, POLY 27%, *p* = 0.130). A comparison of CON and POLY also showed no significant differences between the groups concerning frequency of at least one follow-up with vitamin deficiency (vitamin A: CON 25%, POLY 19%, *p* = 0.369; vitamin B_12_: CON 6%, POLY 0%, *p* = 0.064; vitamin D: CON 81%, POLY 83%, *p* = 0.763; vitamin E: CON 2%, POLY 0%, *p* = 0.307; folic acid: CON 21%, POLY 27%, *p* = 0.344).

A gender-specific analysis of women, who represent the majority in our cohort, revealed similar results compared with the total cohort concerning prevalence of vitamin deficiencies. Vitamin B_12_ was the only vitamin with significant differences between CON_Women_ and PD_Women_ (CON_Women_ 6%, PD_Women_ 0%; *p* = 0.015).

## Discussion

This analysis reveals (i) that psychiatric disease indicated by concomitant intake of psychopharmacological medication is highly prevalent in patients after bariatric surgery and (ii) that patients on psychopharmacological medication who take part in a structured postoperative care program are not at an elevated risk for vitamin deficiencies in long-term follow-up after bariatric surgery. According to the Austrian’s depression report [20], about 10% of the total Austria’s population were taking antidepressants in 2015. This rate was stable over the years 2013 to 2015. The prevalence of patients taking antidepressants in our cohort is approximately two times higher. Further, (iii) patients taking psychopharmacological medication have no adverse long-term outcomes regarding changes in body-mass-index.

However, subtle increases of TG and HbA1c were observed in patients with psychopharmacological medication, which could in part be attributed to drug side-effects or slightly older age. Nevertheless, TG and HbA1c still were in the normal range in this group. A subgroup analysis of patients taking one psychopharmacological drug compared with patients taking multiple psychopharmacological drugs revealed no difference in the prevalence of vitamin deficiencies.

Vitamin deficiencies are a common phenomenon among patients with severe obesity with an indication for bariatric surgery [21]. Life-long vitamin supplementation after bariatric surgery is crucial for favorable long-term outcomes and for prevention of adverse effects. Depending on surgical procedure, vitamin deficiencies are a common phenomenon [22]. Patients with Roux-en-Y gastric bypass are more likely to suffer from vitamin B_12_ deficiency compared with those after sleeve gastrectomy [23]. In our cohort vitamin B_12_ deficiency at each individual visit during observation period was prevalent in only 6% in CON and 1% in PD. This prevalence is lower compared with previous observations at other centers. Other studies reported vitamin B_12_ deficiency in up to 12% of patients [24]. These divergent results might be partly explained by different reference values and surgical procedures performed. The present data is in line with a report by Javanainen et al. who observed a prevalence of vitamin B_12_ deficiency in 1 to 3% of patients 2 years after bariatric surgery when using a comparable reference value (138 pmol/l) [25].

Vitamin D deficiency is a common state not only in patients following bariatric surgery but also in general population with highly variable prevalence ranging from 20 to 80% depending on geographical area [26, 27]. In our cohort 53% of both groups were deficient in vitamin D at every individual visit during the observation period. This high percentage might be explained by the rather high reference value of 75 nmol/l used. Studies with the same reference values of vitamin D reported about 30% of patients reaching sufficient vitamin D levels at 2 years follow-up [25]. Another study using a reference value of 50 nmol/l showed a deficiency rate of 32 to 52%, depending on surgical method [26]. To address the ongoing debate of which vitamin D level should be considered sufficient, we also analyzed the impact of a lower reference value on frequency of deficiency in our cohort. Most of the guidelines consider vitamin D levels more than 50 nmol/l as sufficient and levels of lower than 30 nmol/l as clear deficiency with a state of insufficiency in-between [28]. Nevertheless, a sub-analysis with cut-off values of 30 nmol/l and 50 nmol/l revealed that the proportion of patients being insufficient or deficient in vitamin D was not significantly different between CON and PD.

The measurement of folic acid in serum has been suggested to be a sensitive marker of compliance regarding the regular intake of multivitamin supplements [29]. In our cohort 6% of CON and 4% of PD presented with folic acid levels below 9.53 nmol/l at each individual follow-up, which was considered insufficient. Other studies reported prevalence of folic acid deficiency up to 25%, depending on surgical procedure [30, 31]. Thus, the low percentage of folate deficiency in our cohort, especially in the PD group, likely indicates good adherence to recommended daily multivitamin supplementation.

Vitamin A plays an important role for eye health and deficiencies in vitamin A levels may lead to loss night blindness. It also plays a crucial role in pregnancies after bariatric surgery [32]. In 8% of CON and 9% of PD, vitamin A deficiency was observed at every follow-up visit. Other studies reported similar rates of vitamin A deficiency of 5–12% after bariatric surgery depending on follow-up time [33, 34].

Vitamin E deficiency was very uncommon, as 98% in both groups were within normal reference values of vitamin E at every measurement during the observation period. Other data also suggests that vitamin E deficiency is rarely observed in clinical practice, since most supplements contain vitamin E [35].

Aaseth et al. reported that about 52–83% of patients are taking supplements 5 years after gastric bypass surgery [36]. This finding highlights the importance of adequate counseling regarding adherence to supplementation after bariatric surgery. Of note, in our analysis we assessed the frequencies of recommendations and prescriptions for vitamin/mineral supplementation, which did not differ significantly between CON and PD. Based on the vitamin levels, it can just be assumed that the adherence to recommended vitamin supplementation is not impaired in patients with prescribed psychopharmacological medication. Problems with compliance to scheduled follow-up visits are known to be associated with different weight loss after bariatric surgery [37]. Regarding adherence to follow-up visits in our study, a greater proportion of PD (112 /132, 85%) had more than one follow-up, compared with CON (307/392, 78%). This finding was not associated with significantly different weight loss between these groups.

The major limitation of this study is its retrospective design with all well-known disadvantages. Moreover, in part due to data protection rules, we had no access to detailed psychiatric diagnoses and course of disease. Additionally, there was no detailed information available when intake of psychopharmacological drugs was initiated and stopped. Detailed information on preoperative medication was not available; wherefore, we cannot define the exact onset of psychiatric disease. Postoperative prescription of antipsychotics and antidepressants was observed in 5% and 20%, respectively. These rates are similar to an Austrian-wide registry study including data of 9151 patients who underwent bariatric surgery. The preoperative prescription rate of antidepressants (24%) and antipsychotics (7%) was lower than the postoperative (28% and 9%) [38]. All bariatric surgery candidates at our department have to obtain a psychological clearance before surgery to exclude patients in an unstable condition. Prior studies have noted the high prevalence of psychiatric comorbidities in bariatric surgery candidates [39]. The prevalence of patients taking psychopharmacological medication in our analysis was 25%, which is lower than previously reported frequencies from other studies [40–43]. This might indicate a possible selection bias which could contribute to the good adherence to supplements in our cohort. Some patients with severe psychiatric symptoms might be underrepresented in our study population. There might also be some patients with underlying psychiatric disorders in the control group, which were not detected due to missing therapy or due to non-adherence to psychopharmacological therapy. Non-adherence to psychotropic medication is reported to be high among patients with psychiatric disorders [19].

On the other hand, a strength of this study is the sample size of more than 500 patients and the long mean follow-up time of approximately 5 years, which therefore offers the opportunity to investigate a large patients’ collective in a real-world setting at an obesity outpatients’ clinic. Additionally, the structured assessment of vitamin levels at each individual follow-up adds knowledge about the course of frequencies of vitamin deficiencies. The findings of this analysis might be of practical relevance not only for teams involved in aftertreatment after bariatric surgery but also for the preoperative decision-making process in bariatric surgery candidates with psychiatric disorders and/or concomitant psychopharmacological medication.

Taken together, we conclude that patients with psychiatric disorders who follow the recommended psychopharmacological regimen and follow-up visits after bariatric surgery, are not at higher risk to suffer from vitamin deficiencies compared with controls.
